# Immunocytochemical examination of Akt, mTOR, and Pax-2 for endometrial carcinoma through thin-layer endometrial cytology

**DOI:** 10.3389/fmed.2025.1576060

**Published:** 2025-04-28

**Authors:** Ke Ma, Xi Yang, Zihui Yang, Yiting Meng, Jia Wen, Rui Chen, Jianghui Yang, Qinping Liao

**Affiliations:** ^1^Department of Obstetrics and Gynecology, Beijing Tsinghua Changgung Hospital, School of Clinical Medicine, Tsinghua Medicine, Tsinghua University, Beijing, China; ^2^Institute for Intelligent Healthcare, Tsinghua University, Beijing, China; ^3^Department of Pathology, Beijing Tsinghua Changgung Hospital, School of Clinical Medicine, Tsinghua University, Beijing, China

**Keywords:** immunocytochemical expression, Akt, mTOR, Pax-2, endometrial carcinoma, thin-layer endometrial preparation, endometrial cytology test

## Abstract

**Objective:**

This study aimed to detect the expression of Akt, mTOR, and Pax-2 in differing endometrial tissue/cells, together with assessment of such molecular markers for improving accuracy within endometrial cytology screening for endometrial cancer (EC).

**Methods:**

Overall, 92 hysteroscopy cases were included. This cohort comprised 32 endometrial carcinoma patients, 30 benign lesion patients, and 30 cases with normal endometrium. Endometrial cells were collected before hysteroscopy, for immunohistochemical (IHC) and immunocytochemical (ICC) detection of Akt, mTOR, and Pax-2. Expression levels were evaluated by semi-quantitative method, and ROC curves (Receiver Operating Characteristic Curve) were drawn to evaluate the application importance for all three biomarkers for EC diagnostics.

**Results:**

IHC expression of Akt, mTOR, and Pax-2 was positively correlated with ICC expression within the endometrial carcinoma cohort, benign lesion cohort, and normal cohort. Using IHC and ICC, Akt/mTOR-marked upregulation was observed within the EC cohort, compared to all other cohorts. Pax-2 was also markedly upregulated within normal/benign lesion cohorts in comparison to the EC cohort (*p* < 0.01). The sensitivity and specificity of ICC was 73.33 and 91.53%, respectively, when Akt ≥ 190 was used as the diagnostic index for EC. When mTOR ≥ 255 was used as the diagnostic index for EC, such parameters were 84.38 and 95.00%, respectively. When Pax-2 ≤ 165 was used as the diagnostic index for EC, such parameters were 96.67 and 80.00%, respectively.

**Conclusion:**

This investigation probed varying threshold levels pertaining to Akt, mTOR, and Pax-2, consequently assisting in endometrial lesion-type identification. IHC within ECT (endometrial cytology test) analyses for Akt, mTOR, and Pax-2 could enhance the capacity for diagnosing EC/pre-malignant lesions.

## Introduction

Endometrial carcinoma (EC) ranks as the sixth most prevalent cancer among women globally and is the leading malignancy of the female reproductive tract in developed regions such as the United States, Europe, and economically advanced Chinese cities such as Beijing, Shanghai, and Zhongshan (1, 2). Currently, EC accounts for nearly half of all newly diagnosed gynecologic cancers, with its global incidence having surged by 132% over the past three decades. Notably, EC was increasingly affecting younger women, with the incidence in those under 40 years of age doubled, marking a 100% increase. The lifetime risk for women developing EC is estimated to be approximately 3%. EC presents a significant public health challenge requiring urgent attention in both developed countries and economically advanced cities in China. There is an urgent need to establish effective screening and early diagnostic methods to ensure the timely detection and treatment of EC. Currently, the clinical diagnosis of EC predominantly depends on identifying clinical symptoms, complemented by imaging tests such as gynecologic ultrasound and pelvic magnetic resonance imaging (MRI). Diagnosis is further supported by sampling procedures, including hysteroscopy, curettage, and endometrial cell sampling (3–12).

Our team has dedicated significant attention to endometrial cytology, which has demonstrated high levels of accuracy and sensitivity (13, 14). This approach was recommended in the Expert Consensus on Screening and Early Diagnosis of Endometrial Cancer (Draft) in China in 2017 (15). The endometrial cytology test (ECT) serves as a screening methodology for EC, especially in relevant high-risk cases (13, 14, 16–21). The ECT screening program developed by our research team demonstrated high sampling satisfaction, diagnostic accuracy, and strong negative predictive value. Additionally, liquid-based ECT is an ideal screening tool due to its reasonable cost, high patient acceptance, and ease of use in outpatient settings (13). We have been continuously seeking and exploring methods to enhance diagnostic sensitivity. It is worth further investigating whether specific molecular markers can be added to improve the accuracy of the identification of the ECT.

The PI3K/Akt/mTOR signaling axis is a commonly exploited signal transduction pathway in EC, and its activation is associated with the progression of the disease and poor prognostic outcomes. Pax-2 (22, 23), a member of the extensive paired box gene family, is commonly used as a marker for Müllerian duct derivatives. As a transcription factor, Pax-2 binds to DNA and is exclusively found in the nucleus. The abnormal loss of nuclear Pax-2 expression is observed in approximately 70–80% of cases of EC and atypical hyperplasia/endometrial intraepithelial neoplasia (AH/EIN) (24). This loss represents an early event—possibly even the initiating event—in the progression of AH/EIN. However, the underlying mechanisms behind this loss are not well understood, given that Pax-2 is not among the genes frequently mutated in endometrial carcinoma. It is speculated that the loss may result from epigenetic changes, similar to the suppression of Pax-2 observed during later stages of embryonic development. Numerous studies have validated the diagnostic value of Pax-2 loss in identifying atypical hyperplasia/endometrial intraepithelial neoplasia (AH/EIN).

In this study, immunohistochemistry (IHC) and immunocytochemistry (ICC) were utilized to examine the expression profiles of Akt, mTOR, and Pax-2 expression within normal endometrium, benign lesions, and EC. Moreover, the study evaluated the potential of these three molecular markers to enhance the accuracy of ECT screenings for EC.

## Materials and methods

### Patients

This is a prospective case–control study conducted using a consecutive inclusion program for sample selection. It included 92 patients who underwent segmental diagnostic curettage or simultaneous hysteroscopy for suspicious endometrial lesions in the Department of Obstetrics and Gynecology of Beijing Tsinghua Changgung Hospital from March 2016 to March 2020. Before the operation, all patients signed an informed consent form and underwent endometrial cytology sampling simultaneously, which is routinely performed at our hospital. The study was approved by the Ethics Committee of Beijing Tsinghua Changgung Hospital (No. 17134-0110). All methods were carried out in strict accordance with relevant guidelines and regulations.

Endometrial cell collection was conducted using the SAP-1 endometrial cell collector (Saipujiuzhou, Beijing, China) before hysteroscopy and segmental curettage. This procedure aimed to produce fluid-based cell smears from the endometrium. The expression of Akt, mTOR, and Pax-2 was detected using IHC and ICC, and the results were compared with histopathological and cytopathological diagnoses.

Participants’ median age was 49 years (spanning 24–84 years). This study included a cohort of 32 patients with EC and pre-malignant lesions, including 23 individuals with EC (three patients with G3, 11 with G2, and nine with G1), three patients with clear-cell carcinoma, four with serous papillary carcinoma, and two with endometrial atypical hyperplasia. In the benign proliferative lesion cohort, there were 30 patients, including six cases of simple endometrial hyperplasia, two cases of complex endometrial hyperplasia, and 22 endometrial polyp cases. The normal cohort consisted of 30 cases, including 17 proliferative endometrium, eight secretory endometrium, and five atrophic endometrium patients, respectively.

Indications for hysteroscopic surgery include (1) individuals at high risk for EC, such as those with diabetes mellitus, hypertension, family history of neoplasia, polycystic ovary syndrome, etc.; (2) abnormal uterine bleeding; (3) abnormal sonographic findings, which may be observed on ultrasound, iodine-oil angiography of the fallopian tubes, CT, or MRI; (4) physiologic or specific alterations in the endometrium resulting from hormone replacement or the application of TAM; (5) cervical cytology revealing abnormal intrauterine cells; (6) a histologic diagnosis of a previous abnormal endometrial pathology; (7) secondary dysmenorrhea; and (8) infertility and family planning issues.

### Endometrial cytology

#### Qualified and unqualified specimens of endometrial liquid-based cytology

Inclusion criteria for liquid-based endometrial cytology specimens are as follows: Satisfactory endometrial cytology specimens should meet the conditions of clear labeling, accurate and complete clinical information, and sufficient number of well-preserved endometrial glandular epithelial cells, including at least five to six stacks of endometrial cells, except in cases of atrophic stage endometrium. Any specimen containing abnormal cells will also be considered satisfactory. The exclusion criteria are specified as follows: Unsatisfactory cytology specimens will be determined by one of the following: lack of clear labeling, irreparably broken slides, excessive overlapping of cells, blood, inflammatory cells overlying the endometrial cells, poor fixation, over-drying, contamination, etc. For a specimen to be classified as unsatisfactory, these issues must obstruct the observation of more than 75% of the glandular cells.

The preparation protocol for thin-layer cell smears using the AISO™ liquid-based cytology system (Lan Ang Co. Ltd., Shanghai, China) involves the following steps: (1) Add 1 mL of filling liquid to the film maker, ensuring that the liquid is properly distributed beneath the filter membrane; (2) vigorously shake the bottle five times and pour the contents into the sample; (3) insert the sample into the turntable and activate the automatic production function for 1 min; (4) producer is extracted, upper-tube is rotated by 90°, slide is removed, and bottom-bracket discarded; and (5) rinse the prepared slides with clean water three times and then proceed with staining.

**Figure 1:**
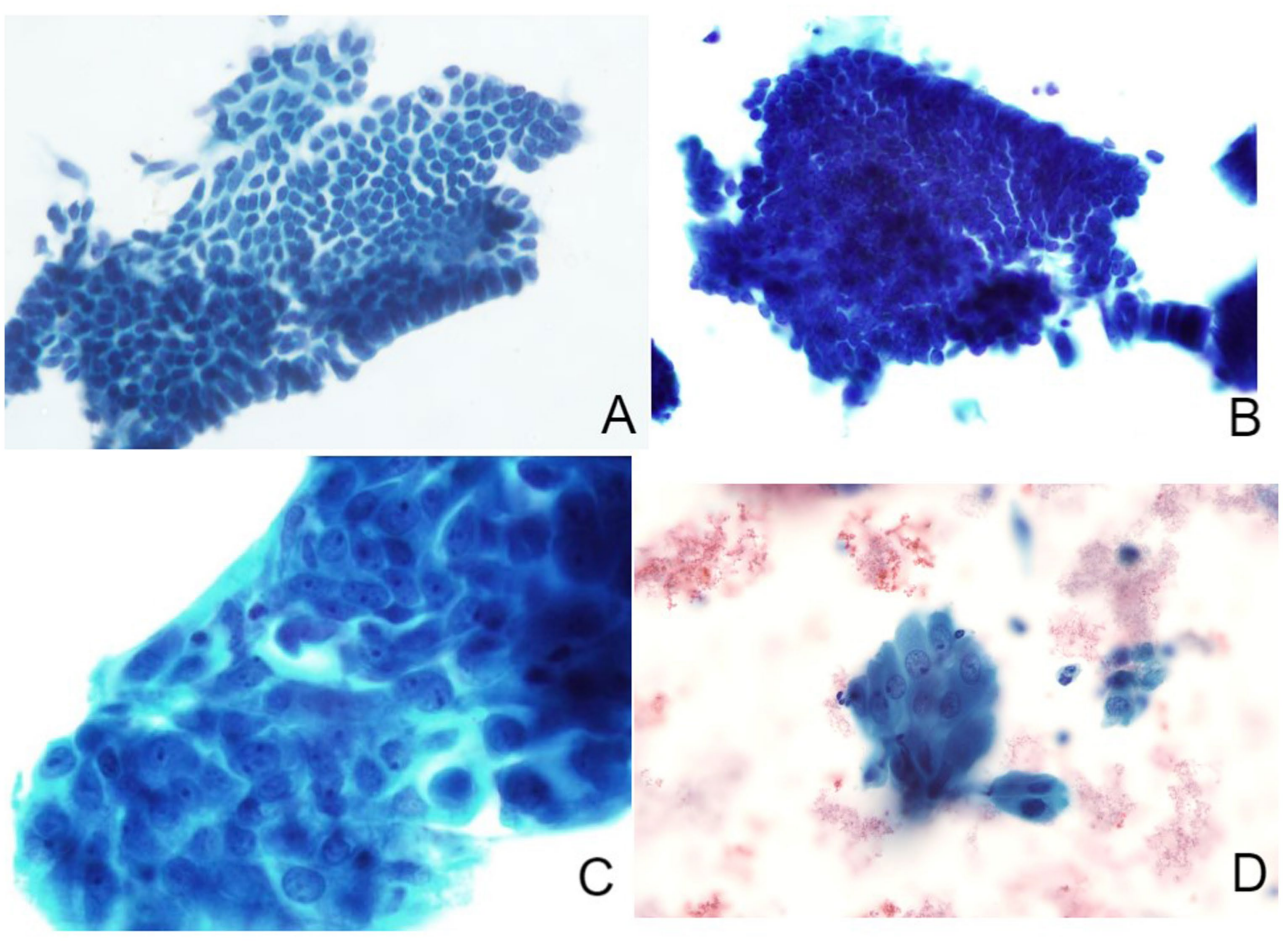
Liquid-based thin-layer cell smears of endometrium (Papanicolaou staining). **(A)** Normal endometrium: cells are neatly organized within single-layer, featuring oval or round nuclei with regular inter-nuclear spaces (magnification: 200×); **(B)** Benign hyperplastic endometrium: cells densely arranged in a monolayer with delicate chromatin and small nucleoli (200×); **(C)** Atypical endometrial cells: intercellular spaces appear heterogeneous, with areas of crowded or overlapping and rough chromatin (600×); **(D)** Suspected EC: cells of varying sizes display prominent round nucleoli, an enlarged nucleoplasm, and vacuoles within the cytoplasm (600×).

The liquid-based thin-layer cell smears, along with histopathological sections, are reviewed by a pathology professional. The diagnostic criteria for endometrial cytology are categorized into the following four groups: (1) no intraepithelial lesions or malignant cells (includes proliferative, secretory, and atrophic membrane cells); (2) benign proliferative shifts (such as simple hyperplasia, complex hyperplasia, irregular proliferation, and endometrial polyps); (3) atypical endometrial hyperplasia; and (4) suspected EC (25) (Figure 1). Histopathological diagnosis is based on the 2003 WHO diagnostic criteria (26).

#### IHC detection

Paraffin-embedded specimens were sectioned at a thickness of 4 μm, with five slices cut from each specimen: one for blank control, one for hematoxylin-eosin staining (H-E) and three for IHC staining.

The paraffin sections were deparaffinized and hydrated using a series of graded ethanol solutions. Consequently, these sections were rinsed with phosphate-buffered saline (PBS) three times before being blocked with 30% peroxide-methanol at room temperature. The assays were conducted in a humidity-controlled environment as follows: (1) sections were incubated with ethylenediaminetetraacetic acid (EDTA) (pH8.0); (2) sections were rinsed three times with PBS; (3) sections underwent overnight incubation with rabbit anti-human AKT antibody (#9611, Cell Signaling Technology™, USA, 1:600 dilution), rabbit anti-human mTOR-related protein antibody (#2983, Cell Signaling Technology™, USA, 1:150 dilution), and rabbit anti-human Pax-2 antibody (ZSGB-BIO™, Beijing, China); (4) sections were rinsed four times with PBS (5 min per rinse); (5) ChemMate™ EnVision™/HRP (Dako™, Herndon, VA, USA) was introduced, followed by incubation of the sections for 30 min at 37°C; (6) sections were rinsed four times with PBS; (7) staining was performed using 3,3-diaminobenzidine (DAB) at room temperature in the dark for 180 s; (8) distilled water halted the DAB staining; (9) sections were hematoxylin-stained; and (10) sections were dehydrated, cleared, and mounted with neutral-gum, with positive controls treated in the same manner.

#### ICC detection

In brief, fixed cytological-imprint smears (CS) were fixed using 95% alcohol, followed by immersion in 50% alcohol and 2×-distilled water for 5 min each. CS was then treated with distilled water. Consequently, the paraffin sections were rinsed with PBS for three cycles of 3 min each and blocked with 3% peroxide-methanol at room temperature to endogenous peroxidase activity. Subsequently, the sections were again rinsed with PBS three times before undergoing a 30% peroxide-methanol block at room temperature. The subsequent assays were conducted within a humidity-controlled environment as follows: (1) sections were incubated with EDTA at 60°C for 10 min; (2) sections were rinsed with PBS three times; (3) sections underwent overnight incubation with rabbit anti-human AKT antibody (#9611, Cell Signaling Technology™, USA, 1:600 dilution), rabbit anti-human mTOR-related protein antibody (#2983, Cell Signaling Technology™, USA, 1:150 dilution), and rabbit anti-human Pax-2 antibody (ZSGB-BIO™, Beijing, China); (4) sections were rinsed four times with PBS (2 min per rinse); (5) ChemMate™ EnVision™/HRP (Dako™, Herndon, VA, USA) was introduced, followed by incubation of the sections for 30 min at 37°C; (6) sections were rinsed four times with PBS; (7) staining was performed using 3,3-diaminobenzidine (DAB) at room temperature in the dark for 180 s; (8) distilled water halted the DAB staining; (9) sections were hematoxylin-stained; and (10) sections were dehydrated, cleared, and mounted with neutral-gum, with positive controls treated in the same manner. Negative controls underwent identical processing, though without primary antibody steps.

Akt, mTOR, and Pax-2 expression profiling depended upon the stain intensity (i) in conjunction with the percentage (%) of positive cells (pi) in whole-tissue sections. Akt, mTOR, and Pax-2 staining were assessed through:

$H-\mathrm{score}=\mathrm{pi}\;\left(i+1\right)\times 100$

This was determined by multiplying pi (0–100%) by corresponding i (0 = negative, 1 = weak, 2 = moderate, and 3 = strong).

#### Statistical analyses

SPSS 22.0 (IBM Corp., Armonk, NY, USA) was utilized to perform all such evaluations. Spearman’s correlation was used to determine the associations between the dysregulation biomarkers (Akt, mTOR, and Pax-2) in IHC and ICC. Kruskal–Wallis test was employed to evaluate the associations between biomarker dysregulation (Akt, mTOR, and Pax-2) and clinicopathological characteristics. The ROC curve was used to assess the diagnostic value for all investigated biomarkers pertaining to EC diagnostics. Links across differing ICC scorings/clinic-pathology-based characteristics were also analyzed.

## Results

### Correlation of Akt, mTOR, and Pax-2 expression in endometrial tissues and cells

**Figure 2:**
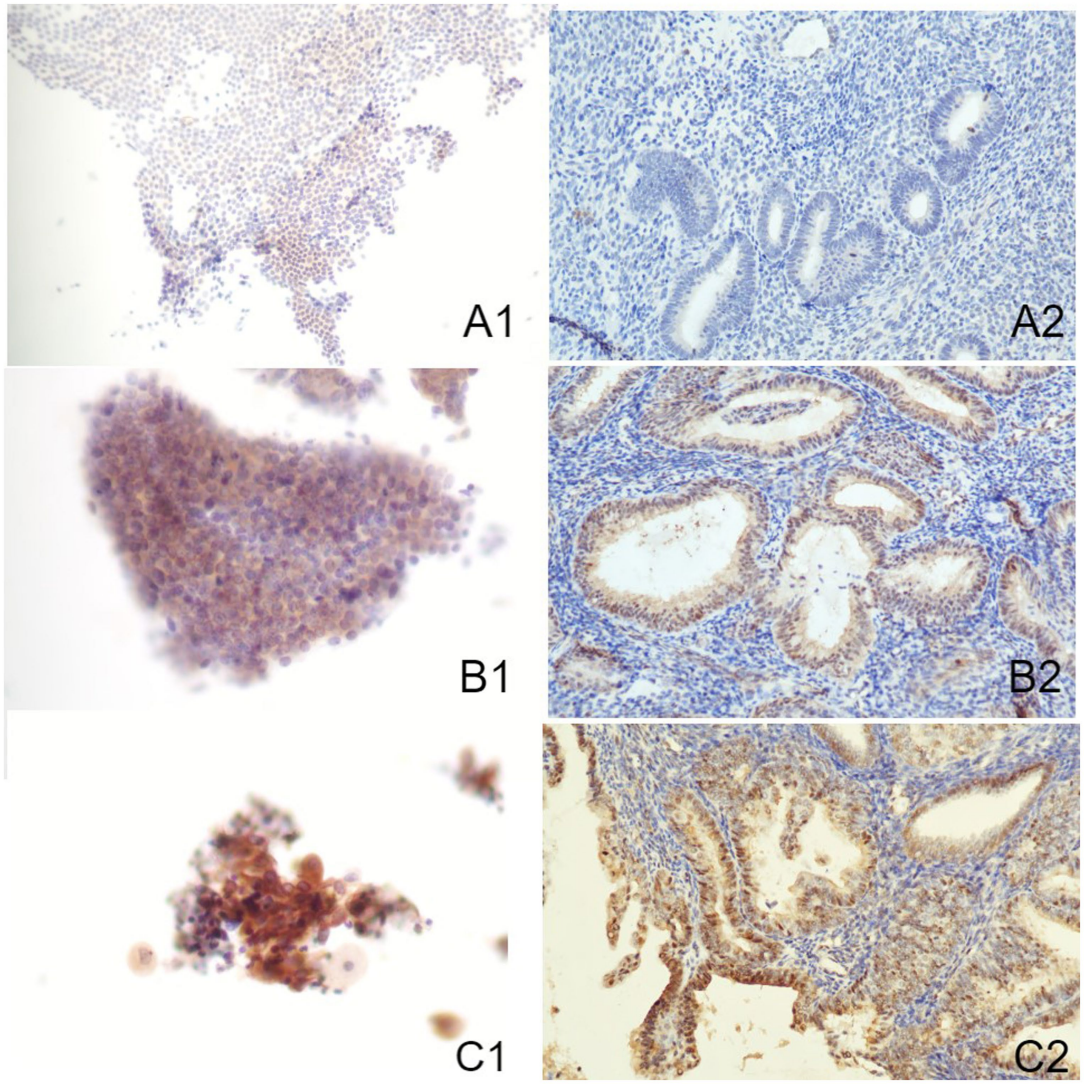
Immune-reactions of Akt in ICC and IHC (Papanicolaou stain, at an original magnification of 20×). Akt was predominantly expressed within nuclei, manifesting as a brownish-yellow granular positive stain. **(A1)** Normal endometrium evaluated by ICC. **(A2)** Normal endometrium evaluated by IHC. **(B1)** Benign endometrial abnormality evaluated by ICC. **(B2)** Benign endometrial abnormality evaluated by IHC. **(C1)** EC evaluated by ICC. **(C2)** EC evaluated by IHC. IHC, immunohistochemistry; ICC, immunocytochemistry; EC, endometrial carcinoma.

**Figure 3:**
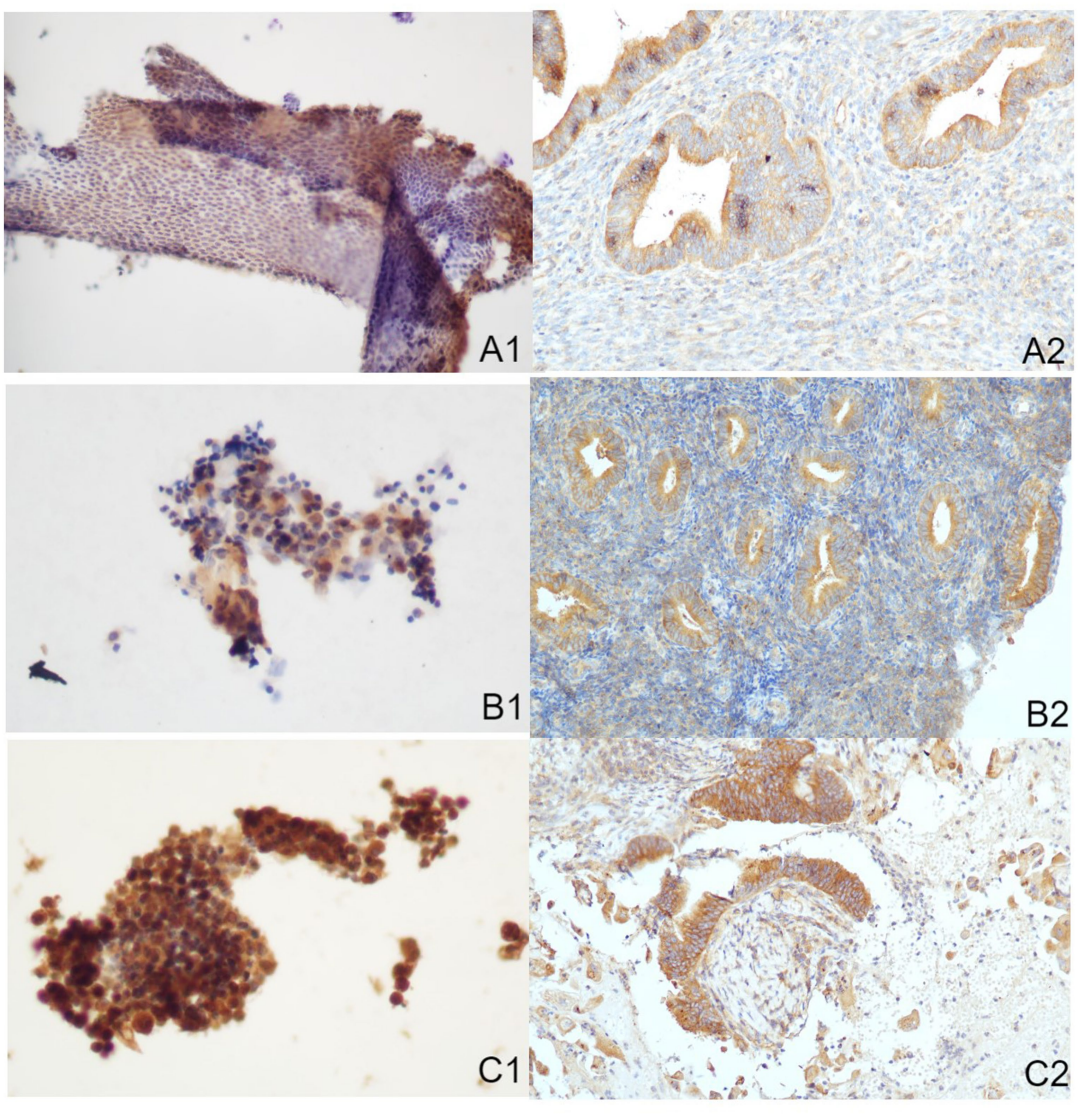
Immune-reactions of mTOR in ICC and IHC (Papanicolaou stain, at an original magnification of 20×). mTOR was predominantly expressed within cytoplasm, manifesting as a brownish-yellow granular positive stain. **(A1)** Normal endometrium identified through ICC. **(A2)** Normal endometrium identified through IHC. **(B1)** Benign endometrial abnormality identified through ICC. **(B2)** Benign endometrial abnormality identified through IHC. **(C1)** EC identified through ICC. **(C2)** EC identified through IHC. IHC, immunohistochemistry; ICC, immunocytochemistry; EC, endometrial carcinoma.

**Figure 4:**
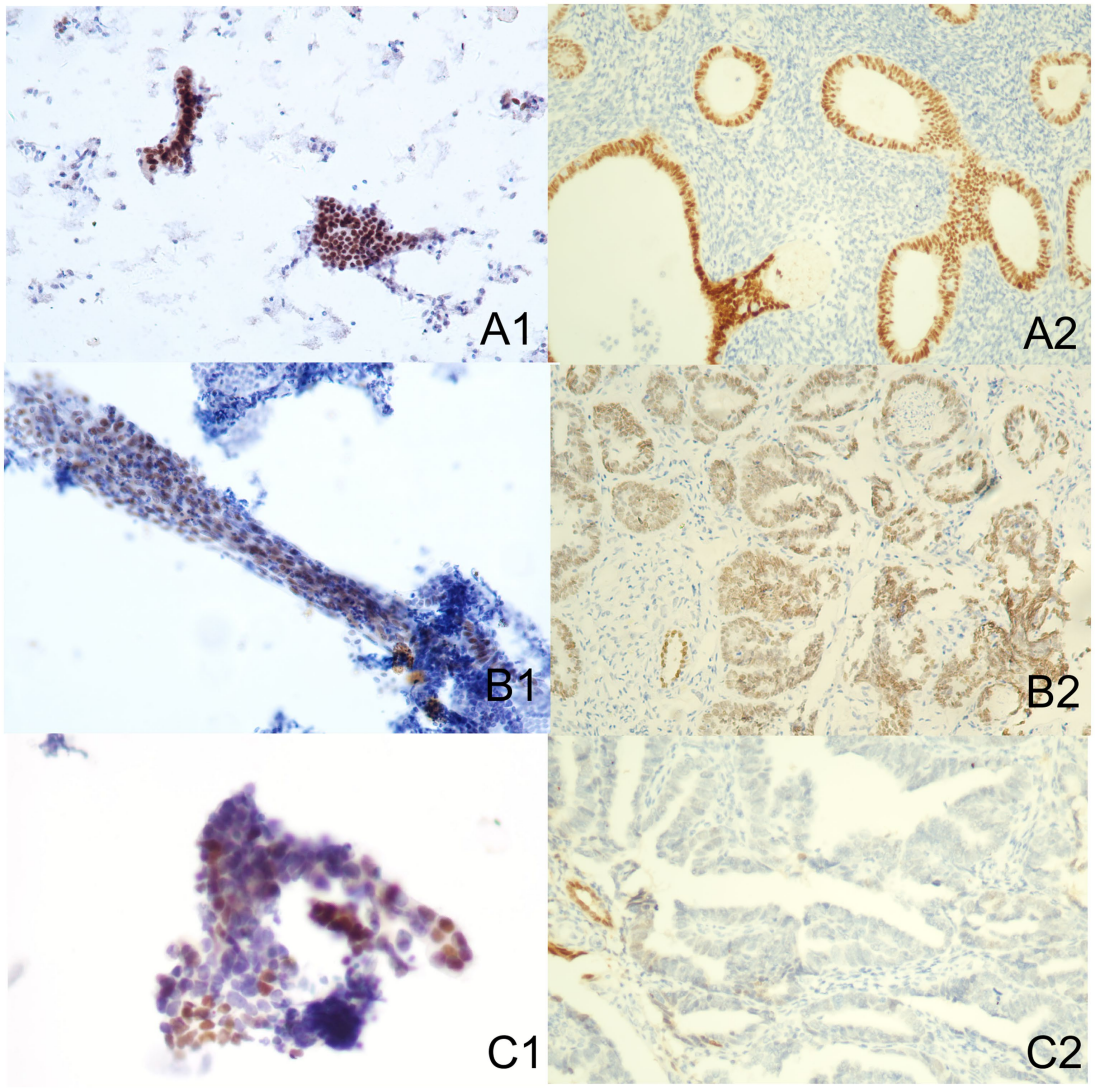
Immune-reactions of Pax-2 in ICC and IHC (Papanicolaou stain, at an original magnification of 20×). Pax-2 was predominantly expressed within nuclei, manifesting as a brownish-yellow granular positive stain. **(A1)** Normal endometrium performed by ICC. **(A2)** Normal endometrium performed by IHC. **(B1)** Benign endometrial abnormality performed by ICC. **(B2)** Benign endometrial abnormality performed by IHC. **(C1)** EC performed by ICC. **(C2)** EC performed by IHC. IHC, immunohistochemistry; ICC, immunocytochemistry; EC, endometrial carcinoma.

**Figure 5:**
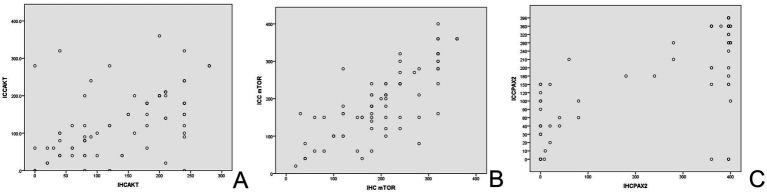
The scatterplot of biomarkers in IHC and ICC. **(A)** Scatterplot for AKT endometrial expression. **(B)** Scatterplot for mTOR endometrial expression. **(C)** Scatterplot for Pax-2 endometrial expression. IHC, immunohistochemistry; ICC, immunocytochemistry.

The Akt protein is expressed in both cytoplasm and nucleus of EC cells and endometrial stromal cells, with positive staining appearing brown-yellow (Figure 2). Furthermore, the mTOR protein is mainly located in cytoplasm (Figure 3). Pax-2 is found in endometrial adenocytes and interstitial cells, especially within nuclei (Figure 4). The stain-intensity of Akt, mTOR, and Pax-2 was evaluated by quantifying the percentage (%) of positive cells together with H-Score determination. The semi-quantitative results of Akt, mTOR, and Pax-2 in both IHC and ICC are shown in Table 1. The Spearman rank correlation analysis demonstrated correlation coefficients (rs) of Akt, mTOR, and Pax-2 to be 0.567, 0.831, and 0.679, respectively, and accordingly, all *p* < 0.001 (Figure 5). There was positive correlation between Akt, mTOR, and Pax-2 expression in IHC and ICC within EC cohort, benign lesion cohort, and normal cohort. In summary, ICC detection can accurately reflect expression for all three such biomarkers within differing endometrial tissues.

**Table 1:** Expression of Akt, mTOR and Pax-2 in IHC and ICC.

Biomarkers	Methods	N	Mean	Min.	Max.	Percentile		
						25	50	75
Akt	IHC	92	146.52	0	280	80	180	210
	ICC	92	138.76	0	360	60	140	200
mTOR	IHC	92	214.02	20	360	160	210	320
	ICC	92	206.52	20	400	150	210	295
Pax-2	IHC	92	239.62	0	400	0	360	396
	ICC	92	199.42	0	396	40	200	360

IHC, immunohistochemistry; ICC, immunocytochemistry; N, numbers of samples; Min., minimum; Max., maximum.

### Comparison of the expression of Akt, mTOR, and Pax-2 in normal, benign, and EC cohorts

Table 2 and Figures 2–4 show the expression of Akt, mTOR, and Pax-2 in different lesion classifications, as determined by IHC and ICC methods. The expression patterns of Akt and mTOR, as indicated by rank means, generally exhibit an increasing trend corresponding to the severity of endometrial lesions, progressing from normal endometrium to benign endometrial abnormality and further to atypical hyperplasia and EC in both IHC and ICC analyses (*p* < 0.01). Conversely, Pax-2 demonstrates a decreasing trend with lesion severity in IHC and ICC analyses. For example, in IHC, the rank mean decreases from 65.28 in normal endometrium to 19.73 in atypical hyperplasia and EC, which is statistically significant (*p* < 0.01). The Akt, mTOR, and Pax-2 show statistically significant differences in expression across different lesion classifications, with *p*-values less than 0.01.

**Table 2:** Expression of Akt, mTOR and Pax-2 in each group of ICC and IHC.

Biomarkers	Lesion classification	IHC				ICC			
		N	Rank mean	*χ* ^2^	*p*	N	Rank mean	*χ* ^2^	*p*
Akt	Normal endometrium	30	28.41	18.31	<0.01	30	26.28	38.04	<0.01
	Benign endometrial abnormality	30	51.02			30	41.05		
	Atypical hyperplasia and EC	32	55.02			32	67.05		
mTOR	Normal endometrium	30	29.40	29.00	<0.01	30	20.63	55.39	<0.01
	Benign endometrial abnormality	30	43.62			30	36.27		
	Atypical hyperplasia and EC	32	65.23			32	70.97		
Pax-2	Normal endometrium	30	65.28	51.60	<0.01	30	66.97	49.14	<0.01
	Benign endometrial abnormality	30	51.48			30	48.90		
	Atypical hyperplasia and EC	32	19.73			32	20.63		

*χ2, Chi-square value; p, p-value; N, numbers of samples. IHC, immunohistochemistry; ICC, immunocytochemistry.*

### The clinical performance of Akt, mTOR, and Pax-2

**Figure 6:**
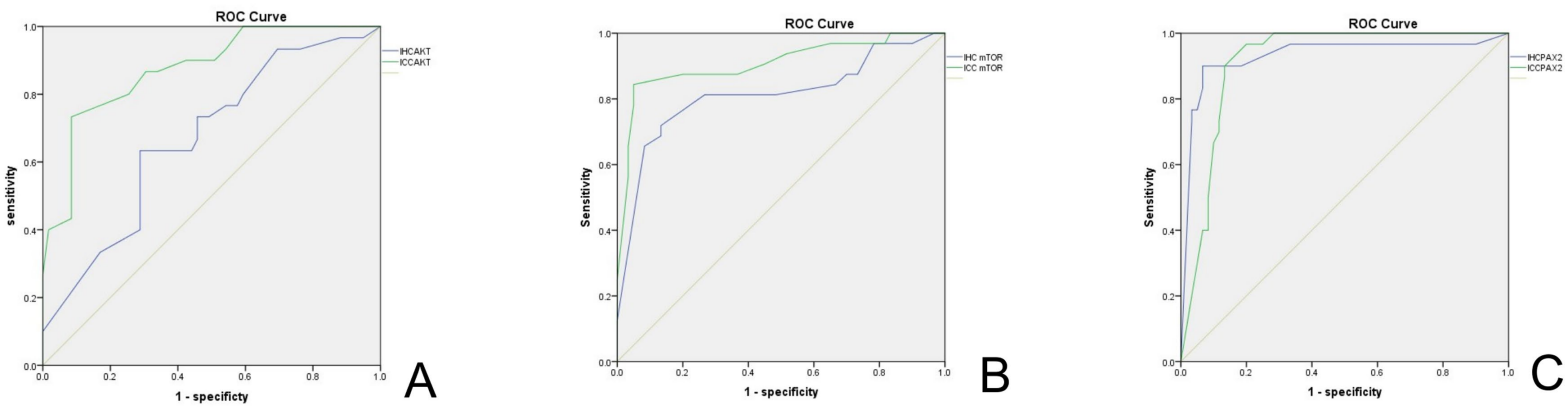
ROC curve of biomarkers in IHC and ICC. **(A)** ROC curve for AKT. **(B)** ROC curve for mTOR. **(C)** ROC curve for Pax-2. The green line indicates ICC, and the blue line represents IHC. IHC, immunohistochemistry; ICC, immunocytochemistry.

Figure 6 illustrates the ROC curves for the biomarkers Akt, mTOR, and Pax-2 as evaluated through IHC and ICC. Table 3 and Supplementary Tables 1–3 present a comparative analysis of the clinical performance of Akt, mTOR, and Pax-2 biomarkers using IHC and ICC, evaluating sensitivity, specificity, Youden’s index, and area under the curve (AUC) at ideal thresholds to determine their diagnostic efficacy. In IHC, Pax-2, with an ideal threshold of ≤50.00, demonstrates superior accuracy, featuring an AUC of 0.93 (95%CI, 0.86–0.99), a sensitivity of 90.00%, a specificity of 93.33%, and a Youden’s index of 0.83, surpassing the performance of Akt and mTOR. In ICC, mTOR, with an ideal threshold of ≥255, and Pax-2, with ideal thresholds of ≤135 and ≤165, exhibit a high AUC of 0.91. Akt and mTOR demonstrate superior specificity, exceeding 90% at 91.53 and 95.00%, respectively, higher than Pax-2’s specificity of above 80.00%.

**Table 3:** The clinical performance of Akt, mTOR, and Pax-2in IHC and ICC.

Biomarkers	IHC					ICC				
	Ideal threshold	Sen. (%)	Spe. (%)	Youden’s index	AUC (95%CI)	Ideal threshold	Sen. (%)	Spe. (%)	Youden’s index	AUC (95%CI)
Akt	≥145	73.33	54.24	0.28	0.65 (95%CI, 0.54–0.78)	≥190	73.33	91.53	0.65	0.87 (95%CI, 0.80–0.95)
mTOR	≥255	71.88	86.67	0.59	0.81 (95%CI, 0.70–0.91)	≥255	84.38	95.00	0.79	0.91 (95%CI, 0.83–0.98)
Pax-2	≤50	90.00	93.33	0.83	0.93 (95%CI, 0.86–0.99)	≤135	90.00	86.70	0.77	0.91 (95%CI, 0.86–0.97)
						≤165	96.67	80.00	0.77	0.91 (95%CI, 0.86–0.97)

IHC, immunohistochemistry; ICC, immunocytochemistry; Sen., sensitivity; Sep., specificity; AUC, area under the ROC curve.

## Discussion

Endometrial carcinoma (EC) ranked as the sixth most prevalent malignant tumor in women worldwide and is the leading gynecologic malignancy in developed regions, including the United States, Europe, and cities such as Beijing, Shanghai, and Zhongshan in China (1, 2). Over the past three decades, the global incidence of EC has surged by 132%, with the condition now accounting for nearly 50% of all new gynecologic cancer diagnoses. Notably, EC is increasingly affecting younger women, with cases among those under 40 years of age doubling in number, reflecting a 100% rise. The lifetime risk of developing EC in women is approximately 3%.

In economically advanced areas of China, the incidence of EC has been on the rise. According to data from the National Cancer Center of China in 2024, approximately 77,000 new EC cases are reported annually, equating to an incidence rate of 11.25 per 100,000 people (27). This marks a significant increase from 2004, when the rate was 6.51 per 100,000, representing more than a doubling of the incidence over a 20-year period. Data from the Beijing Tumor Registry Office indicated that since 2001, EC incidence has surpassed that of cervical cancer. By 2008, EC became the most common malignant tumor in the female reproductive system, with an incidence rate of 21.76 per 100,000 by 2018.

Recent economic growth in China has led to lifestyle and dietary changes, which have contributed to an increase in metabolic diseases. These conditions are strongly associated with the rising and younger incidence of EC. Key risk factors for EC include obesity, diabetes, a history of estrogen or tamoxifen use, polycystic ovary syndrome, and infertility—conditions commonly linked to the lifestyle in developed nations. Given these trends, screening for EC is vital, especially for asymptomatic women at high risk. In both developed countries and economically prosperous cities in China, EC represents a critical public health issue that demands urgent attention. Effective screening and early diagnostic strategies are essential for the timely detection and treatment of EC.

Currently, the clinical diagnosis of EC relies primarily on clinical symptoms, imaging techniques such as gynecologic ultrasound and pelvic MRI, and invasive procedures such as hysteroscopy, curettage, and endometrial cell sampling (3–12). However, these methods have certain limitations: while ultrasound and pelvic MRI are highly sensitive, they lack specificity. Invasive procedures, including hysteroscopy, tissue biopsy, and curettage, can cause psychological distress and discomfort for patients. Our research team has concentrated on endometrial cytology, showing high accuracy and sensitivity in diagnostic applications (13, 14). In fact, it was recommended in the 2017 Expert Consensus on Screening and Early Diagnosis of Endometrial Cancer (Draft) in China (15). Our study has been cited in Japan’s endometrial cancer screening guidelines (28), where endometrial cancer screening has been legally mandated. Since the implementation of Japan’s Elderly Health Care Law in 1987, a population-based screening model has been in place, with more than 200,000 individuals participating annually. Of those screened, approximately 2% require further treatment, and the diagnosis rate exceeds 0.1%.

According to the Expert Consensus on Screening and Early Diagnosis of EC, released in 2017 in China, routine screening should not be implemented within the intermediate-risk EC population, though screening is recommended for the high-risk EC population (29). Several guidelines recommend transvaginal ultrasound (TVS) as the preferred method of screening for EC. However, TVS has reduced specificity, positive predictive value, and an elevated false-positive rate (5, 6). Most guidelines recommend using disposable endometrial samplers for EC biopsy (3, 5, 6, 8, 30–32), and the preparation method after sampling is endometrial cytology (3, 32). Previously, the widespread use of endometrial cytology smears has been hindered by the fact that smears often contain a large amount of blood cells, mucus, and excessive cellular overlap, since endometrial pathology is complex. Since 1996, liquid-based cytology (LBC) has received approval through the U.S. Food and Drug Administration (FDA) to be the primary method of cervical cancer screening. LBC greatly improved specimen quality. During the past decade, LBC has also been widely used in endometrial cell smears (13, 14, 17–21, 33–38). Studies have shown that diagnosis-linked sensitivity, specificity, and positive/negative predictive values for EC screening by endometrial fluid-based cytology are 78–100%, 66–100%, 41.9–100%, and 96–100%, respectively (13, 14, 16–21). It should be noted that another major advantage of endometrial fluid-based cytology is the extremely low incidence of insufficient specimen collection (2.2–4%), especially in the postmenopausal population. Compared with endometrial biopsy, cytology has a higher sampling satisfaction rate (up to 96.8%) (13, 14). Endometrial cytology has received increasing attention and recognition (3, 32). The review of “Endometrial Cancer” published in ‘The Lancet’ in 2022 recognized the accuracy of endometrial cytology (1). The 2020 Japanese Oncology Guidelines include endometrial cytology as an EC screening methodology, citing our previous research (32).

However, interpreting endometrial cytology presents significant challenges due to the influence of the menstrual cycle and the varied morphological characteristics of the endometrium. Differentiating between proliferative stage endometrial cells and highly differentiated endometrial carcinoma cells can be particularly complex. Moreover, the glandular morphology adds another layer of difficulty when analyzing cytological samples. As a result, training experts to accurately interpret endometrial cytology demand considerable time, resources, and effort. Therefore, we hope to improve the accuracy of endometrial cancer by assisting endometrial cytology through immunocytochemistry and, even more, to develop sophisticated kits for clinical application.

In addition to providing cytopathologic diagnosis, the remaining specimens can be tested for molecular biology. Whether ECT can improve the accuracy of EC screening by detecting specific biomarkers is worthy of further study. PI3K/Akt/mTOR pathway is the most valuable cellular signaling pathway, playing a role within multiple physiological and pathological processes, and can regulate several downstream target genes that inhibit cellular apoptosis and increase cellular proliferation (39). Studying target molecules in this transduction pathway has become a hot spot in tumor research. Akt is a molecule directly acting downstream of PI3K. Following activation of the signal transduction pathway, the conformation of Akt is changed, and subsequent phosphorylation occurs, activating Akt. Activation of PI3K/Akt/mTOR thwarts apoptosis driven through various triggers, promoting cell-cycle advancement, thereby enhancing cellular survival/proliferative properties, and participating in angiogenesis, having pivotal parts within tumorigenesis, also exacerbating tumor invasiveness/metastases. Activated Akt triggers mTOR and downstream TSC1 and TSC2 to regulate cell growth and protein translation. MTOR, a downstream molecule in the PI3K/Akt/mTOR axis, serves as a master regulating player for cellular development. It not only plays a major role in normal cell proliferation but also participates in the regulation of various physiological processes that are closely related to tumorigenesis, such as cell growth and proliferation, cell cycle regulation, and cell migration (40). The Pax-2 gene is a subtype of the PAX gene family. Transcription factors encoded by the Pax-2 gene not only have a decisive effect on important organ development, such as the genitourinary system and central nervous system, but also have a close relationship with the occurrence of various organ-based malignant tumors. It is believed that the loss of Pax-2 gene expression—as an early event—implicates it within EC development, serving as an auxiliary indicator for clinical diagnosis of EC and endometrial dysplasia (41). Therefore, in this study, Akt, mTOR, and Pax-2 were selected as molecular markers for ICC detection, and their application value in improving the accuracy of ECT screening for EC was probed.

In this study, Akt, mTOR, and Pax-2 were detected in ECT smear and paraffin tissue sections for each enrolled subject, and the dataset outcomes demonstrated that Akt, mTOR, and Pax-2 expressions were highly associated (*p* < 0.001), indicating that ICC method in ECT was stable and reliable. Regardless of the IHC or ICC detection method, this study found that Pax-2 was markedly upregulated within the normal/benign lesions cohort in comparison to the EC cohort, while Akt and mTOR were markedly upregulated within the EC cohort in comparison to the remaining cohorts. These results indicated that the application of the above three molecular markers in ECT screening EC is feasible. Such dataset outcomes show that diagnostic sensitivity/specificity could be enhanced when all three molecular markers are added to ECT. In the results of the study, in the case of ICC, the area under the curve of ROC was greater than 0.85, with mTOR and Pax-2 greater than 0.9, showing a high diagnostic value. Apostolou et al. (42), together with Norimatsu et al. (43), suggested a score-based method by merging ICC evaluations for Ki-67/p53 through cytology-based imprints collected from extracted uteri with finalized scorings valuable within diagnoses. Both studies highlighted LBC to have pivotal parts within ICC-based phosphatase and tensin homolog (PTEN), β-catenin, and p53 expression within endometrial carcinoma specimens. We believe that endometrial cytology is an effective and feasible method for screening EC in high-risk patients with EC. In endometrial cytology, immunocytochemical staining can increase the accuracy of screening by utilizing the characteristics of molecular biology that are differentially expressed in cancer and normal tissue. Despite the considerable advancements in immunohistochemical staining for endometrial cytology, the process still heavily relies on the manual interpretation of slides by cytopathologists. This approach is associated with substantial labor costs, prolonged training periods, and challenges in widespread dissemination, limiting its accessibility and efficiency in clinical settings. However, with the rapid and extensive development of artificial intelligence (AI) technologies, our research team is actively exploring the application of AI in automating cytopathology slide analysis. By leveraging AI, we aim to enhance the accuracy of endometrial cytology screenings for endometrial cancer while making the process more efficient, cost-effective, and easily adaptable for broader clinical use. This innovative integration of AI has the potential to revolutionize early detection, improving diagnostic precision, and facilitating the widespread implementation of endometrial cancer screening.

Although the current study was conducted at a single institution, future studies will include multiple centers to improve the external validity of the findings and enhance their general applicability. One limitation of this study is the lack of follow-up data. Future studies will incorporate follow-up data to assess the long-term prognostic value of these molecular markers in endometrial cancer patients. Although we have made efforts to control for potential confounding variables, including age and hormonal levels, future studies will further investigate and adjust for these factors to minimize their potential influence on the results.

Our study highlights the potential clinical applications of Akt, mTOR, and Pax-2 as diagnostic markers in endometrial cancer screening. By enhancing screening accuracy, these molecular markers may play an important role in early diagnosis, prognostic assessment, and personalized treatment strategies for endometrial cancer.

Endometrial cytology screening for EC is in line with the characteristics of screening methods, reasonable cost, high acceptance of patients, and conducive to popularization. Moreover, additional requirements for such screening tools are present within endometrial LBC, including its cost-effectiveness in comparison to liquid-driven Pap testing, high patient compliance, and non-complex employment within the clinical and GP settings. The coming years are deemed to witness EC emerging into a major health issue, with scarce effective diagnostic/prognostic tools for identifying and monitoring patients that develop such tumors, placing them at elevated risks. Together with shifts in lifestyles and avoiding risk factors, the authors believe that established and regulated screens for high-risk women will emerge as a main challenge for the upcoming generation.

## Data Availability

The raw data supporting the conclusions of this article will be made available by the authors without undue reservation.
