# Supplementary material for: Immunocytochemical examination of Akt, mTOR, and Pax-2 for endometrial carcinoma through thin-layer endometrial cytology
Source: Front Med (Lausanne). 2025 Apr 28;12:1576060. doi: 10.3389/fmed.2025.1576060 (PMC12066341; doi:10.3389/fmed.2025.1576060)
Supplement: Supplementary file 1 [file Supplementary_file_1.docx]

**Supplementary**

**Supplementary Table 1.** Sensitivity, Specificity, and Youden's Index for Akt Using Varying Threshold Values in IHC and ICC Analysis

| Methods | Threshold | Sensitivity (%) | Specificity (%) | Youden’s index |
| --- | --- | --- | --- | --- |
| IHC | ≥10.00 | 96.67 | 5.08 | 0.02 |
|  | ≥25.00 | 96.67 | 10.17 | 0.07 |
|  | ≥35.00 | 96.67 | 11.86 | 0.09 |
|  | ≥50.00 | 93.33 | 23.73 | 0.17 |
|  | ≥60.00 | 93.33 | 23.73 | 0.17 |
|  | ≥70.00 | 93.33 | 30.51 | 0.24 |
|  | ≥85.00 | 80.00 | 40.68 | 0.21 |
|  | ≥95.00 | 76.67 | 42.37 | 0.19 |
|  | ≥110.00 | 76.67 | 45.76 | 0.22 |
|  | ≥120.00 | 76.67 | 45.76 | 0.22 |
|  | ≥130.00 | 73.33 | 50.85 | 0.24 |
|  | **≥145.00** | 73.33 | 54.24 | 0.28 |
|  | ≥155.00 | 66.67 | 54.24 | 0.21 |
|  | ≥170.00 | 63.33 | 55.93 | 0.19 |
|  | ≥190.00 | 63.33 | 71.19 | 0.35 |
|  | ≥205.00 | 40.00 | 71.19 | 0.11 |
|  | ≥225.00 | 33.33 | 83.05 | 0.16 |
|  | ≥240.00 | 33.33 | 83.05 | 0.16 |
|  | ≥260.00 | 10.00 | 100.00 | 0.10 |
| ICC | ≥10.00 | 100.00 | 8.47 | 0.09 |
|  | ≥30.00 | 100.00 | 13.56 | 0.14 |
|  | ≥50.00 | 100.00 | 30.51 | 0.31 |
|  | ≥70.00 | 100.00 | 40.68 | 0.41 |
|  | ≥85.00 | 93.33 | 45.76 | 0.39 |
|  | ≥95.00 | 90.00 | 49.15 | 0.39 |
|  | ≥110.00 | 90.00 | 57.63 | 0.48 |
|  | ≥130.00 | 86.67 | 66.10 | 0.53 |
|  | ≥145.00 | 86.67 | 69.49 | 0.56 |
|  | ≥165.00 | 80.00 | 74.58 | 0.55 |
|  | **≥190.00** | 73.33 | 91.53 | 0.65 |
|  | ≥205.00 | 43.33 | 91.53 | 0.35 |
|  | ≥225.00 | 40.00 | 98.31 | 0.38 |
|  | ≥260.00 | 26.67 | 100.00 | 0.27 |
|  | ≥300.00 | 10.00 | 100.00 | 0.10 |
|  | ≥340.00 | 3.33 | 100.00 | 0.03 |
|  | ≥361.00 | 0.00 | 100.00 | 0.00 |

**Supplementary Table 2.** Sensitivity, Specificity, and Youden's Index for mTOR Using Varying Threshold Values in IHC and ICC Analysis

| Methods | Threshold | Sensitivity (%) | Specificity (%) | Youden’s index |
| --- | --- | --- | --- | --- |
| IHC | ≥19.00 | 100.00 | 0.00 | 0.00 |
|  | ≥25.00 | 100.00 | 1.67 | 0.02 |
|  | ≥35.00 | 100.00 | 3.33 | 0.03 |
|  | ≥50.00 | 96.88 | 10.00 | 0.07 |
|  | ≥70.00 | 96.88 | 13.33 | 0.10 |
|  | ≥90.00 | 96.88 | 16.67 | 0.14 |
|  | ≥110.00 | 96.88 | 21.67 | 0.19 |
|  | ≥120.00 | 96.88 | 21.67 | 0.19 |
|  | ≥135.00 | 87.50 | 26.67 | 0.14 |
|  | ≥155.00 | 87.50 | 30.00 | 0.18 |
|  | ≥170.00 | 84.38 | 33.33 | 0.18 |
|  | ≥180.00 | 81.25 | 51.67 | 0.33 |
|  | ≥190.00 | 81.25 | 53.33 | 0.35 |
|  | ≥205.00 | 81.25 | 55.00 | 0.36 |
|  | ≥225.00 | 81.25 | 73.33 | 0.55 |
|  | ≥240.00 | 81.25 | 73.33 | 0.55 |
|  | **≥255.00** | 71.88 | 86.67 | 0.59 |
|  | ≥275.00 | 68.75 | 86.67 | 0.55 |
|  | ≥300.00 | 65.63 | 91.67 | 0.57 |
|  | ≥340.00 | 12.50 | 100.00 | 0.13 |
|  | ≥361.00 | 0.00 | 100.00 | 0.00 |
| ICC | ≥19.00 | 100.00 | 0.00 | 0.00 |
|  | ≥30.00 | 100.00 | 1.67 | 0.02 |
|  | ≥50.00 | 100.00 | 10.00 | 0.10 |
|  | ≥70.00 | 100.00 | 16.67 | 0.17 |
|  | ≥90.00 | 96.88 | 18.33 | 0.15 |
|  | ≥110.00 | 96.88 | 28.33 | 0.25 |
|  | ≥120.00 | 96.88 | 28.33 | 0.25 |
|  | ≥130.00 | 96.88 | 33.33 | 0.30 |
|  | ≥145.00 | 96.88 | 35.00 | 0.32 |
|  | ≥155.00 | 93.75 | 48.33 | 0.42 |
|  | ≥170.00 | 90.63 | 55.00 | 0.46 |
|  | ≥180.00 | 87.50 | 63.33 | 0.51 |
|  | ≥190.00 | 87.50 | 65.00 | 0.53 |
|  | ≥205.00 | 87.50 | 68.33 | 0.56 |
|  | ≥225.00 | 87.50 | 80.00 | 0.68 |
|  | ≥240.00 | 87.50 | 80.00 | 0.68 |
|  | ≥**255.00** | 84.38 | 95.00 | 0.79 |
|  | ≥275.00 | 78.13 | 95.00 | 0.73 |
|  | ≥290.00 | 65.63 | 96.67 | 0.62 |
|  | ≥310.00 | 56.25 | 96.67 | 0.53 |
|  | ≥340.00 | 25.00 | 100.00 | 0.25 |
|  | ≥380.00 | 3.13 | 100.00 | 0.03 |
|  | ≥401.00 | 0.00 | 100.00 | 0.00 |

**Supplementary Table 3.** Sensitivity, Specificity, and Youden's Index for Pax-2 Using Varying Threshold Values in IHC and ICC Analysis

| Methods | Threshold | Sensitivity (%) | Specificity (%) | Youden’s index |
| --- | --- | --- | --- | --- |
| IHC | ≤4.00 | 73.33 | 96.67 | 0.70 |
|  | ≤9.00 | 76.67 | 96.67 | 0.73 |
|  | ≤15.00 | 76.67 | 95.00 | 0.72 |
|  | ≤30.00 | 83.33 | 93.33 | 0.77 |
|  | **≤50.00** | 90.00 | 93.33 | 0.83 |
|  | ≤70.00 | 90.00 | 91.67 | 0.82 |
|  | ≤130.00 | 90.00 | 88.33 | 0.78 |
|  | ≤210.00 | 90.00 | 86.67 | 0.77 |
|  | ≤260.00 | 90.00 | 85.00 | 0.75 |
|  | ≤320.00 | 90.00 | 81.67 | 0.72 |
|  | ≤370.00 | 96.67 | 66.67 | 0.63 |
|  | ≤388.00 | 96.67 | 65.00 | 0.62 |
|  | ≤398.00 | 96.67 | 10.00 | 0.07 |
|  | ≤401.00 | 100.00 | 0.00 | 0.00 |
| ICC | ≤5.00 | 40.00 | 93.30 | 0.33 |
|  | ≤15 | 40.00 | 91.70 | 0.32 |
|  | ≤25 | 43.33 | 91.70 | 0.35 |
|  | ≤35 | 50.00 | 91.70 | 0.42 |
|  | ≤60 | 66.66 | 90.00 | 0.57 |
|  | ≤85 | 70.00 | 88.30 | 0.58 |
|  | ≤95 | 73.33 | 88.30 | 0.62 |
|  | ≤110 | 86.67 | 86.70 | 0.73 |
|  | **≤135** | 90.00 | 86.70 | 0.77 |
|  | ≤165 | 96.67 | 80.00 | 0.77 |
|  | ≤190 | 96.67 | 75.00 | 0.72 |
|  | ≤205 | 100.00 | 71.70 | 0.72 |
|  | ≤225 | 100.00 | 68.30 | 0.68 |
|  | ≤260 | 100.00 | 66.70 | 0.67 |
|  | ≤300 | 100.00 | 55.00 | 0.55 |
|  | ≤340 | 100.00 | 51.70 | 0.52 |
|  | ≤378 | 100.00 | 13.30 | 0.13 |
|  | ≤397 | 100.00 | 0.00 | 0.00 |
